# Immunization of young heifers with staphylococcal immune evasion proteins before natural exposure to *Staphylococcus aureus* induces a humoral immune response in serum and milk

**DOI:** 10.1186/s12917-018-1765-9

**Published:** 2019-01-07

**Authors:** Lindert Benedictus, Lars Ravesloot, Kim Poppe, Ineke Daemen, Eveline Boerhout, Jos van Strijp, Femke Broere, Victor Rutten, Ad Koets, Susanne Eisenberg

**Affiliations:** 1 Department of Infectious Diseases and Immunology, Faculty of Veterinary Medicine, Utrecht University, Utrecht, The Netherlands; 2 Division of Infection and Immunity, The Roslin Institute, The University of Edinburgh, Easter Bush, Midlothian, EH25 9RG Scotland, UK; 3 Department of Large Animal Health, Faculty of Veterinary Medicine, Utrecht University, Utrecht, The Netherlands; 4 Department of Bacteriology and Epidemiology, Wageningen Bioveterinary Research, Lelystad, The Netherlands; 5 Ruminants Research and Development, MSD Animal Health, Boxmeer, The Netherlands; 6 Department of Medical Microbiology, University Medical Center Utrecht, Utrecht, The Netherlands; 7 Department of Veterinary Tropical Diseases, Faculty of Veterinary Science, University of Pretoria, Onderstepoort, South Africa; 8 Niedersächsische Tierseuchenkasse, Hanover, Germany

**Keywords:** *Staphylococcus aureus*, Mastitis, Experimental immunization, Natural exposure, Non-protective immunity, Milk antibodies, LukM, Efb, Cattle

## Abstract

**Background:**

*Staphylococcus aureus*, a leading cause of mastitis in dairy cattle, causes severe mastitis and/or chronic persistent infections with detrimental effects on the cows’ wellbeing, lifespan and milk production. Despite years of research there is no effective vaccine against *S. aureus* mastitis. Boosting of non-protective pre-existing immunity to *S. aureus*, induced by natural exposure to *S. aureus,* by vaccination may interfere with vaccine efficacy. The aim was to assess whether experimental immunization of *S. aureus* naïve animals results in an immune response that differs from immunity following natural exposure to *S. aureus*.

**Results:**

First, to define the period during which calves are immunologically naïve for *S. aureus,* Efb, LukM, and whole-cell *S. aureus* specific serum antibodies were measured in a cohort of newborn calves by ELISA. Rising *S. aureus* specific antibodies indicated that from week 12 onward calves mounted an immune response to *S. aureus* due to natural exposure. Next, an experimental immunization trial was set up using 8-week-old heifer calves (*n* = 16), half of which were immunized with the immune evasion molecules Efb and LukM. Immunization was repeated after one year and before parturition and humoral and cellular immunity specific for Efb and LukM was determined throughout the study. Post-partum, antibody levels against LukM and EfB were significantly higher in serum, colostrum and milk in the experimentally immunized animals compared to animals naturally exposed to *S. aureus*. LukM specific IL17a responses were also significantly higher in the immunized cows post-partum.

**Conclusions:**

Experimental immunization with staphylococcal immune evasion molecules starting before natural exposure resulted in significantly higher antibody levels against Efb and LukM around parturition in serum as well as the site of infection, i.e. in colostrum and milk, compared to natural exposure to *S. aureus*. This study showed that it is practically feasible to vaccinate *S. aureus* naïve cattle and that experimental immunization induced a humoral immune response that differed from that after natural exposure only.

**Electronic supplementary material:**

The online version of this article (10.1186/s12917-018-1765-9) contains supplementary material, which is available to authorized users.

## Background

Intramammary infections with *Staphylococcus aureus* (SA) are common in cattle and often lead to severe mastitis and/or chronic persistent infections with detrimental effects on the cows’ wellbeing, lifespan and milk production [1, 2]. The pathogenesis of *S. aureus* mastitis involves the attachment of *S. aureus* to epithelial cells [3], followed by the production of a range of immune evasion molecules which severely impede effective targeting of *S. aureus* by the immune system [4–8]. In addition, cell invasion and biofilm formation decreases antibiotic effectivity, resulting in partial clearance and increased antibiotic resistance [4, 9, 10]. The ineffective treatment of *S. aureus* mastitis often leads to chronic infections, therefore, prevention of *S. aureus* infection by vaccination is highly desirable [11, 12]. However, despite numerous attempts and the use of various vaccine antigens, todays available vaccines against *S. aureus* only result in limited protection [13–15]. Pre-existing immunity may influence the response to vaccination [16–18]. The majority of dairy cattle have pre-existing immunity against *S. aureus* at first calving due to natural exposure to *S. aureus* during rearing (e.g. through (transient) colonization), as evidenced by high antibody titers against *S. aureus* and several of its secreted immune evasion proteins [19–21]. The existing immune response against *S. aureus* seems to be non-protective, since infection with *S. aureus* does not protect against subsequent infections [22–25]. We therefore hypothesize that vaccination of non-naïve cows may lead to boosting of the existing non-protective immune response rather than the induction of a protective immune response. Understanding the dynamics of immunity induced following natural exposure to *S. aureus* in young calves will help to determine the period during which dairy calves are still naïve for *S. aureus*.

The aim of the present study was to investigate whether it is feasible to vaccinate *S. aureus* naïve animals and whether experimental immunization of *S. aureus* naïve animals results in a different immune response compared to immunity induced by natural exposure to *S. aureus*. We have previously reported a high prevalence of antibodies against the *S. aureus* immune evasion proteins extracellular fibrinogen-binding protein (Efb) and the leukocidin subunit LukM in dairy cows [19]. Efb forms a shield of host proteins around the bacterium, helping *S. aureus* escape from phagocytic cells [26], while LukM is the receptor binding subunit of the bi-component leukocidin LukMF’, a potent toxin with an important role in *S. aureus* mastitis [27–29]. First, to determine the time period when *S. aureus* naïve animals can be vaccinated the kinetics of maternal immunity and the onset of immunity against *S. aureus* following natural exposure were studied by measuring Efb, LukM and whole-cell *S. aureus* specific antibodies in a cohort of new-born calves. Secondly, young calves were immunized with an experimental vaccine containing recombinant Efb and LukM, prior to the development of immunity to *S. aureus* due to natural exposure, and the development of humoral and cellular immune responses were measured for two years up until the first post-partum period. These responses were compared to the immune responses following natural exposure to *S. aureus*. Experimental immunization starting before natural exposure to *S. aureus* resulted in significantly higher antibody levels against Efb and LukM around parturition in serum as well as in colostrum and milk, i.e. at the site of infection, compared to natural exposure.

## Methods

### Study design

All procedures and treatments were approved by the Ethical Committee for Animal Experiments of Utrecht University (Permit No. DEC0202080601 and Permit No. 2012.II.09.136) and performed according to national and European regulations. After the described studies, all animals were kept alive and either joined the teaching dairy herd, were reared until sent for slaughter or were reused for other animal experiments.

To study antibody dynamics due to natural exposure to *S. aureus* and its secreted evasion proteins (Study 1), 22 pregnant Holstein-Friesian (cross) heifers, purchased from commercial farms or reared at the research facility, were housed at the research facilities of the Faculty of Veterinary Medicine, Utrecht University (Utrecht, The Netherlands) in 2004. Heifers were housed in tie-stalls on rubber mats covered with sawdust and were fed according to their requirements with roughage and concentrates, while water was available ad libitum. General health was inspected on a daily basis. Heifers were observed by camera to monitor for signs of calving. All calvings were supervised and assisted when necessary. Calves were separated from their dams directly after birth to collect a pre-colostral blood sample. Calves were fed colostrum from their own dam after the first blood sampling. The first colostrum feeding was performed as soon as the calf was able to stand and swallow. Subsequent feedings were performed at 6–8 h intervals and aimed to feed calves a total of 4–6 l of colostrum during the first 24 h by spontaneous uptake.

For the experimental immunization study (Study 2) 16 Holstein-Friesian or Holstein-Friesian cross heifer calves were purchased from commercial farms with a mean age of three weeks (+/− 1 week) in autumn 2013. Calves were housed in groups of four animals on straw bedding at the Faculty of Veterinary Medicine and fed milk replacer, concentrates and roughage according to their requirements. In Spring/Summer 2014 and 2015 heifers were kept as a herd on pasture. In autumn, winter and spring heifers were housed in tie-stalls and were enrolled in a timed artificial insemination protocol to synchronize gestation. In the autumn of 2015 the heifers calved at the research facility. Heifers were observed during calving and assisted when necessary. Colostrum was milked from each quarter of the dam and a mixed colostrum sample was stored at − 20 °C until further analysis. General health was inspected on a daily basis. Around three months of age all animals had mild respiratory problems, six animals were treated with NSAID’s. Around 14 months of age all animals had medium to severe respiratory problems. All animals were treated with NSAID’s and seven animals were treated with antibiotics adhering to national good veterinary practice protocols on the use of antibiotics.

At the start of the experimental immunization trial, heifer calves were assigned to one of four treatment groups (Table 1) using block randomization. After an acclimatization period of two to three weeks animals were immunized at six weeks of age (+/− 1 week) according to the scheme in Table 1, with an experimental vaccine containing the recombinant secreted immune evasion proteins LukM and Efb (Prime). Three booster immunizations were administered, the first six weeks after prime (B1), the second 52 weeks later (B2) and the third two weeks before the expected calving date (B3), which was around 99 weeks after the prime. Subcutaneous (SC) immunization was carried out in the midline of the inguinal region at the future location of the udder using a 2 mL syringe (Omnifix, Braun, Melsungen, Germany) with a 21G needle (Terumo Europe N.V., Leuven, Belgium). Intranasal (IN) application was performed using a nasal spray pump directly into the nostril which produced an aerosolized inoculum with a wide droplet size range. Adjuvant (Adj) only vaccine was administered intranasally at Prime and subcutaneously at the same site as for the SC group for the booster immunizations.

**Table 1:** Experimental immunization scheme of study 2

	Prime (Week 0)	B1 (Week 6)	B2 (Week 52)	B3 (~Week 101)
Group 1 (*n* = 4)	SC	SC	SC	SC
Group 2 (*n* = 4)	IN			
Group 3 (*n* = 4)	IN	IN	Adj (SC)	Adj (SC)
Group 4 (*n* = 4)	Adj (IN)	Adj (SC)		

B1, B2, B3 – Booster immunization 1, 2, 3; SC – Subcutaneous route; IN – Intranasal route; Adj – Adjuvant only immunization

The study was originally designed to compare the immune response following both IN and SC immunization to immunity following natural exposure to *S. aureus*. However, analysis of immune responses following the first booster immunization showed that there was no detectable antibody response following IN immunization (Additional file 1). In a concurrent study where cows were immunized with the same experimental vaccine there was also no detectable antibody response following IN immunization [19]. Antibody and cytokine responses between the IN/IN and Adj/Adj groups and between the IN/SC and SC/SC groups were comparable at week 7 (Additional file 2). Therefore, the IN immunization route was dropped and from B2 onwards groups were combined; heifers of the IN/IN group were added to the Adj/Adj group and received adjuvant only subcutaneously at B2 and B3, while animals of the IN/SC group reacted similarly to the SC/SC group and received the experimental vaccine at B2 and B3 subcutaneously. Leaving only two groups: an experimental immunization group (SC) and a natural exposure control group (Adj) (Table 1). Twelve of the sixteen heifers were successfully inseminated and could therefore be immunized before parturition (B3) (6 Adj; 6 SC). The four non-pregnant animals were removed from the study from week 56 onwards. All data were analyzed according to the experimental immunization and natural exposure control group only, starting from time point zero.

### Vaccine composition and recombinant proteins

The experimental vaccine, consisting of an oil-in-water adjuvant combined with an alginate hydrogel (proprietary adjuvant, MSD-AH), contained 50 μg *S. carnosus* derived recombinant Efb and 50 μg *E. coli* derived LukM per dose. For the prime and first booster immunization (B1) 5 μg Cholera toxin (Sigma-Aldrich Chemie B.V., Zwijndrecht, The Netherlands), a mucosal adjuvant, was added to enhance mucosal immunity [30]. Since no intranasal (i.e. mucosal) immunization was carried out at immunizations B2 and B3, Cholera toxin was not included.

Recombinant Efb and LukM expressed in *E. coli* were generated as described previously [19] and were used for all assays. At week 0 recombinant Efb protein was not available and could, therefore, not be used in the cell based assays in that week. To produce the *S. carnosus* derived Efb used for the vaccine, the gene encoding *efb* from the *S. aureus* Newbould305 strain (ATCC29740) was amplified by PCR and ligated into a pXR100 derived vector and transfected into *S. carnosus*. *S. carnosus* culture supernatant was filtered with a 0.2 μm filter, analyzed on gel for Efb purity and concentration, and stored at − 20 °C.

### Sample collection and preparation

In study 1 colostrum and serum was collected directly after parturition of enrolled heifers and of prenatal calves a serum sample was obtained before colostrum was administered. Further serum samples of the calves were taken one week after parturition and at week 5, 12, 23 and 29 of life. In study 2 blood samples were collected before the first immunization (week 0), at week 7, 52, 56, ~ 99 (two weeks before predicted parturition date) and ~ 103 (two weeks after predicted parturition date).

Blood was collected from the jugular vein in animals up to one year of age. In older animals the coccygeal vein was used. A sterile blood collection system was used to collect blood in Li-heparin and serum tubes (Vacutainer, Becton Dickinson or Vacuette, Greiner Bio-One). After coagulation serum samples were centrifuged for 20 min at 1500 x g to collect serum. Colostrum samples were collected directly after calving from all 4 quarters. Milk samples were collected of all 4 quarters at the morning milking. Colostrum and milk samples were centrifuged for 10 min at 1500 x g to obtain skimmed colostrum/milk. Serum, colostrum and milk samples were stored at − 20 until further analysis.

Total white blood cells (WBC) were isolated by lysing erythrocytes using ammonium-chloride-potassium lysis buffer.

### Antibody ELISA

LukM, Efb and *S. aureus* whole-cell specific IgG1 and IgG2 antibodies in serum, colostrum and milk were determined by ELISA. Because *S. aureus* may express Protein A, which leads to non-specific binding of antibodies, the Protein A negative Reynolds strain was used for the whole-cell ELISA [31]. The Reynolds strain was grown in trypticase soy broth at 39,5 °C and after 20 h bacteria were inactivated with 0,5% formalin overnight. For LukM, Efb and *S. aureus* whole-cell antibody detection, microtiter plates (NUNC MaxiSorp™, eBioscience, Affymetrix, Santa Clara, USA) were coated with 1.25 μg/ml, 1.0 μg/ml and 1 μg/well antigen in 0.05 M sodium-bicarbonate buffer, respectively. Plates were blocked using Blocking buffer (‘Blocking reagent for ELISA’, Roche Diagnostics GmbH, Germany), except for the LukM ELISA for study 1 which were blocked in fat free milk powder (Elk melk, FrieslandCampina, Amersfoort, The Netherlands) and for the *S. aureus* whole-cell ELISA blocking was done with 24 μg casein/well. Serum, milk and colostrum samples were tested in duplicate according to the dilutions shown in Additional file 3. Monoclonal mouse anti-bovine IgG1 and IgG2 (Prionics Lelystad B.V., Life Technologies, Thermo Fisher Scientific) were used as secondary antibodies. Bound secondary antibodies were detected using horseradish conjugated goat anti-mouse-IgG (Biolegend, San Diego, USA). Finally, Tetramethylbenzidine (TMB; Pierce™, Life Technologies, Thermo Fisher Scientific) was used as a substrate and reactions were stopped by adding 4 N sulphuric acid. Extinctions (450 nm) were measured on a Multiscan™ FC Microplate Photometer (Thermo Fisher Scientific) within 5–20 min depending on the specific ELISA.

In order to standardize results and to compare results between plates, positive control serum samples were included in quadruplicate in all ELISA’s and sample to positive ratios were calculated as (sample-buffer control)/(positive-buffer control).

### Whole blood stimulation

RPMI 1640 (Gibco), supplemented with Glutamax™, 50 IU/ml Penicillin, 50 μg/ml Streptomycin, 50uM β-mercaptoethanol and 10% FCS, was used as culture medium. 500 μL heparinized blood was added to 500 μL culture medium with antigens, resulting in a final concentration of Efb and LukM of 10 μg/ml. The negative control was culture medium only and Concanavalin A at 2,5μg/ml was used as a positive control. Whole blood was incubated at 37 °C and 5% CO_2_ in a humidified incubator. After 48 or 72 h supernatants were collected from separate assays for the interferon-γ (IFN-γ) and Interleukin-17a (IL17a) ELISA, respectively, and stored at − 20 °C.

### Proliferation

WBC were washed in PBS and subsequently stained with Carboxyfluorescein succinimidyl ester (CFSE; Thermo Fisher Scientific) at 0.625–1.25 μM for 5 min at 37 °C. Stained WBC were washed twice in culture medium and resuspended in culture medium in a volume equal to the original blood volume. Next, 500 μL CFSE stained WBC were added to 500 μL culture medium with or without antigens, as for the whole blood stimulation. After 96 h incubation at 37 °C and 5% CO_2_ in a humidified incubator, cells were harvested, washed in FACS buffer (FB; PBS supplemented with 2% FCS and 0.01% sodium azide) and stained with mouse monoclonal antibodies against bovine CD4 (CC8-Alexa Fluor® 647, AbD Serotec, Kidlington, UK) or with mouse anti-CD8 (CC63-Alexa Fluor® 647, AbD Serotec) and mouse anti-TCR1/N24 (GB21A-Alexa Fluor® 405, VMRD, Pullman, Washington, USA), a gamma delta T-cell marker. The anti-TCR1/N24 antibody was conjugated with Zenon® anti-mouse IgG2b-Alexa Fluor® 405 Fab fragment (Thermo Fisher Scientific). Cells were incubated for 20 min at 4 °C in the dark and washed twice with FB before being acquired on a FACS Canto II (Becton Dickinson Immunocytometry Systems, San Jose, California, USA).

### Intracellular cytokine staining

WBC were washed and resuspended in culture medium in a volume equal to half of the original blood volume. 500 μL WBC suspension was added to 500 μL culture medium with or without antigens, as for the whole blood stimulation. After a six day incubation period, PMA (50 ng/ml, Sigma-Aldrich) and Ionomycin (1 μg/ml, Sigma-Aldrich) were added to the cells. After a further 1 h incubation Brefeldin A (10 μg/mL; Sigma-Aldrich) was added to each well and incubation was continued for another 5 h. Cells were harvested and washed once in FB and divided for intracellular IFN-γ and IL17a staining. First cells were incubated for 20 min at 4 °C in the dark with mouse monoclonal antibodies against bovine CD4 (CC8-Alexa Fluor® 647, AbD Serotec, Kidlington, UK) and CD8 (CC63-PE, AbD Serotec). Cells were washed and fixed and permeabilized using Cytofix/Cytoperm and Perm/Wash method (Becton Dickinson) according to manufacturer’s instructions. Subsequently cells were stained with a biotinylated anti-bovine IFN-γ monoclonal antibody (6C3; BioSource, San Diego, California, USA) or biotinylated anti-bovine IL17a polycolonal antibodies (Kingfisher Biotech, Inc.) for 30 min at 4 °C in the dark. Next, cells were incubated with streptavidin-eFluor450 (eBioscience, Vienna, Austria) for 30 min at 4 °C in the dark. After two washing steps with Perm/Wash solution, samples were resuspended in FB and acquired on a FACS Canto II.

### Cytokine ELISA

Supernatants from the 48 h and 72 h whole blood stimulation assay were analyzed for the presence of interferon-γ (IFN-γ) and Interleukin-17a (IL17a), respectively. IFN-γ was detected using the Bovigam ELISA (Prionics) according to the manufacturer’s protocol. Results were expressed as sample to positive ratio. IL17a was detected by a quantitative ELISA. Microtiter plates (EIA/RIA Costar™) were coated overnight at 4 °C with 2 μg/mL rabbit-α-bovine IL17a polyclonal antibody (Kingfisher Biotech, Inc., Saint Paul, Minnesota, USA) in 50 μl in 0.05 M sodium-bicarbonate buffer. Plates were blocked using Blocking buffer and supernatant samples diluted 1:2 in Blocking buffer were added in triplicate and incubated at room temperature for 2 h. Standard curves of recombinant bovine IL17a protein (Kingfisher Biotech, Inc.), ranging from 15.6–1000 pg/mL, were included in triplicate on each plate. For detection a biotinylated rabbit-α-bovine polyclonal antibody (0.5 μg/mL) was added and incubated for 30 min at room temperature. Bound antibodies were detected using streptavidin-PolyHRP80 (100 ng/mL) for 20 min at room temperature. The ELISA’s were continued as for the antibody ELISA’s. Standard curves were used to calculate the IL17a concentrations from the absorbance values.

### Data analysis and statistics

Flow cytometry data were analyzed using Flowjo software (TreeStar Inc.). Live cells were selected based on a forward sideward scatter gate. Additional file 4 shows the flow cytometry gating strategy. For graphical presentation of the data GraphPad Prism (GraphPad Software Inc. 6.01, La Jolla, USA) was used and all figures show mean with SEM. Proliferation data and data from the whole blood stimulation were normalized using a log transformation. Descriptive- and statistical analyses were performed in Graphpad Prism or Excel (Microsoft office 2010, Microsoft, Redmond, USA) with animal as the experimental unit. Results were analyzed using unpaired two-sided T-tests corrected for unequal variances. To correct for multiple comparisons, *p*-values were adjusted using the step-down Holms-Bonferroni method. Adjusted *P*-values < 0.05 were considered significant. *P*-values < 0.05 that were not significant after the multiple comparisons correction were considered a trend.

## Results

### Dynamics of *Staphylococcus aureus* specific antibodies in newborn calves

**Fig. 1:**
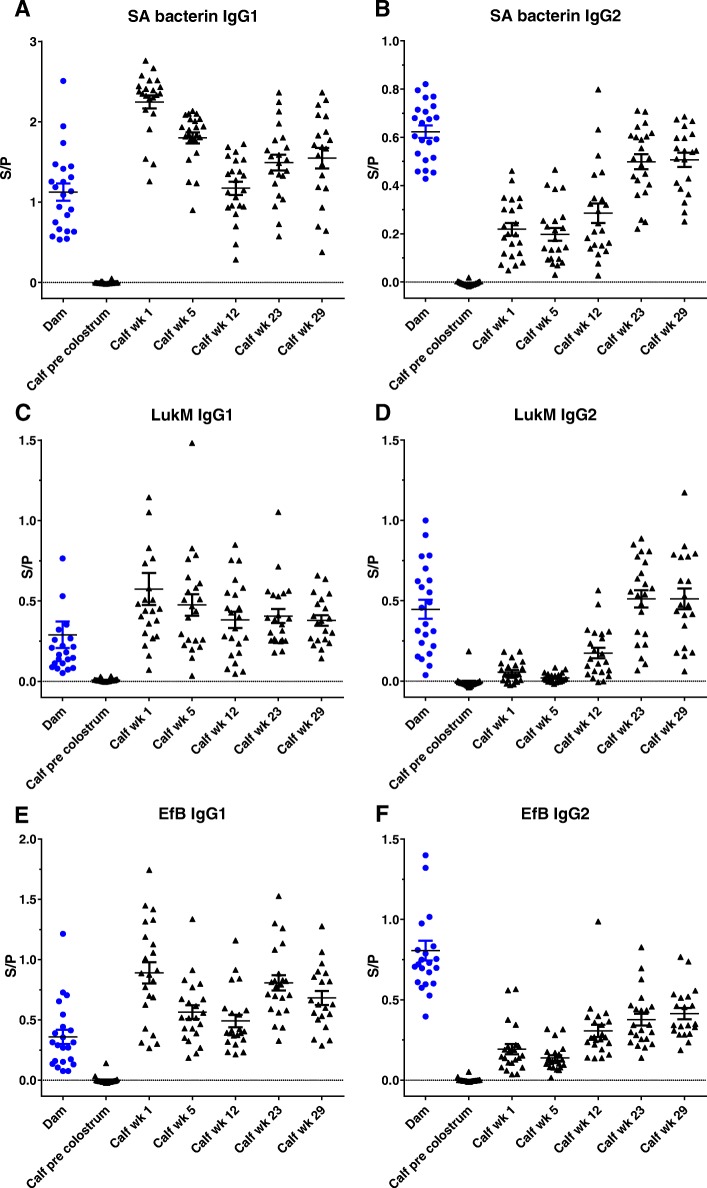
Dynamics of *Staphylococcus aureus* specific antibodies following natural exposure. IgG1 (**a**, **c**, **e**) and IgG2 (**b**, **d**, **f**) antibodies specific for whole SA bacterium (**a**, **b**), LukM (**c**, **d**) and EfB (**e**, **f**) measured in colostrum of dams and in serum of calves from before colostrum uptake until week 29. S/P = Sample to positive ratio

At parturition all dams had detectable IgG1 and IgG2 antibodies against whole-cell *S. aureus* and the secreted proteins LukM and Efb in serum (Additional file 5) as well as colostrum (Fig. 1). Before colostrum intake all calves had virtually no detectable antibodies specific for *S. aureus*, LukM and Efb (Fig. 1). Following absorption of maternal antibodies from colostrum, antibody levels in the calves increased steeply, with serum IgG1 levels exceeding that of their respective dams. The IgG1 concentration in colostrum is much higher than the IgG2 concentration [32] and therefore calf serum IgG2 antibody levels did not increase as much relative to the maternal antibody levels. Colostrum antibody levels correlated reasonably well with dam serum levels, especially for the single antigens, with R^2^s ranging from 0.21–0.85, whereas for whole-cell *S. aureus* the R^2^ was between 0.17 and 0.32 (Additional file 5). As expected, colostrum antibody levels correlated with calf serum antibody levels following colostrum intake, with R^2^s ranging from 0.34–0.79, except for whole-cell *S. aureus* specific IgG1 antibody levels which had and R^2^ of 0.03 (Additional file 5). However, the colostrum IgG1 levels were in the upper range of the ELISA, lowering the correlation.

Once taken up by the calf, maternal antibodies are used and degraded. This was reflected by declining antibody levels in the calves between week 1 and week 12 (Fig. 1), which was most apparent for the IgG1 antibody levels. From week 12 onwards IgG1 and IgG2 levels started rising, which was especially clear for IgG2 due to the low initial IgG2 levels. Overall IgG1 and IgG2 antibodies specific for whole-cell *S. aureus*, LukM and Efb showed the same dynamics. The results indicate that due to natural exposure to *S. aureus* the calves mounted a humoral immune response against *S. aureus* around 12 weeks of age.

### Antibody responses induced by experimental immunization

**Fig. 2:**
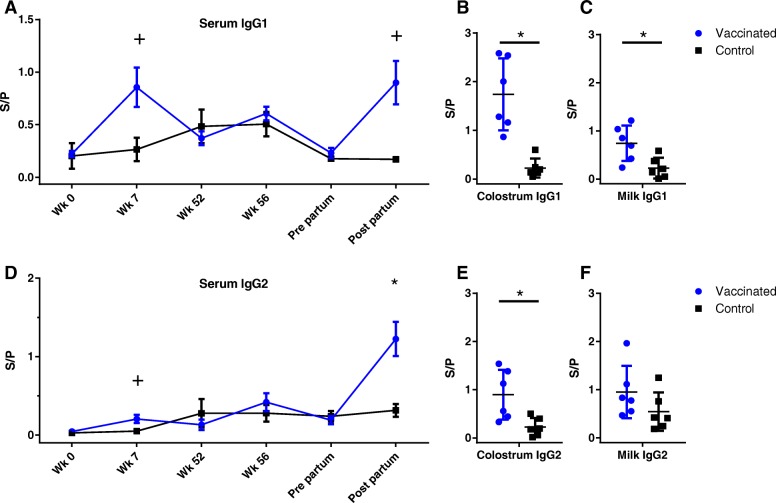
Antibody responses against LukM. IgG1 (**a**, **b**, **c**) and IgG2 (**d**, **e**, **f**) antibodies specific for LukM measured in serum (**a**, **d**), colostrum (**b**, **e**) and milk (**c**, **f**). S/P = Sample to positive ratio. + = *P* < 0,05 before correction for multiple comparisons. * = *P* < 0,05 after correction for multiple comparisons

**Fig. 3:**
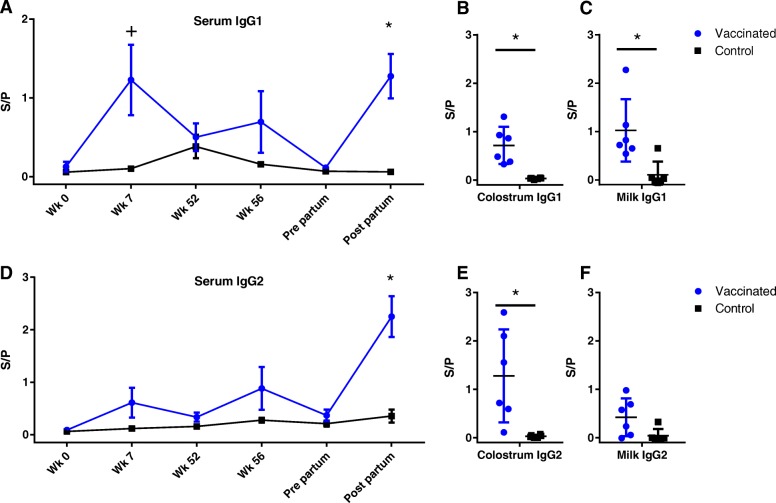
Antibody responses against EfB. IgG1 (**a**, **b**, **c**) and IgG2 (**d**, **e**, **f**) antibodies specific for EfB measured in serum (**a**, **d**), colostrum (**b**, **e**) and milk (**c**, **f**). S/P = Sample to positive ratio. + = *P* < 0,05 before correction for multiple comparisons. * = *P* < 0,05 after correction for multiple comparisons

Serum ELISA pre-immunization showed that the calves enrolled in the experimental immunization study had low antibody levels for LukM and Efb at 8 weeks of age. After the prime and boost immunization antibody levels rose in the experimental immunization group, with a trend for higher levels compared to the natural exposure control group at week 7 for LukM specific IgG1 and IgG2 and Efb specific IgG1 (*P* < 0.05 before multiple comparison correction, Figs. 2 and 3). Whereas the antibody levels dropped between week 7 and 52 in the experimentally immunized animals, antibody levels rose in the control group, presumably because of natural exposure to *S. aureus* between the first and second booster immunization. Following the boost in week 52 antibody levels rose slightly in the experimentally immunized animals, but were not significantly different from the control group. Two weeks post-partum, after the third boost, antibody levels were significantly higher in the experimentally immunized animals for LukM specific IgG2 and Efb specific IgG1 and IgG2 and there was a trend for higher levels for LukM specific IgG1 (Figs. 2 and 3). Antibody levels in colostrum were significantly higher in the experimentally immunized animals for both LukM and Efb specific IgG1 and IgG2 (Figs. 2 and 3). LukM and Efb specific IgG1 antibodies were also significantly higher in milk two weeks post-partum (Figs. 2 and 3).

### Cellular immune responses induced by experimental immunization

**Fig. 4:**
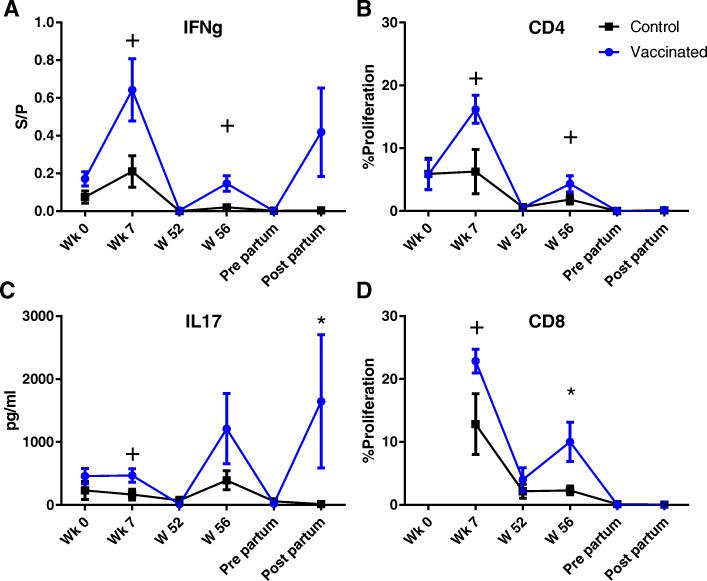
Cellular immune responses following stimulation with LukM. IFNg (**a**) and IL17a (**c**) production following stimulation of whole blood with LukM for 48 h and 72 h, respectively. Proliferation measured as the percentage of CD4 (**b**) and CD8 (**d**) T-cells with diluted CFSE signal following 96 h stimulation with LukM. S/P = Sample to positive ratio. + = *P* < 0,05 before correction for multiple comparisons. * = *P* < 0,05 after correction for multiple comparisons

**Fig. 5:**
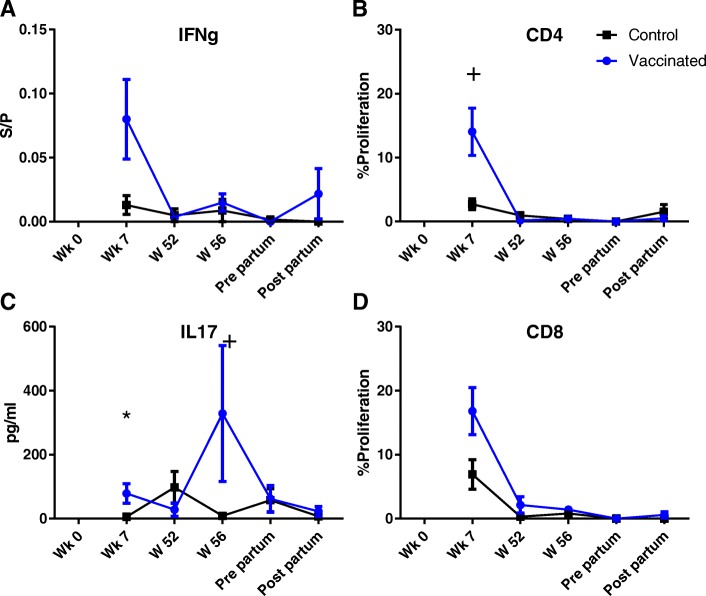
Cellular immune responses following stimulation with EfB. IFNg (**a**) and IL17a (**c**) production following stimulation of whole blood with EfB for 48 h and 72 h, respectively. Proliferation measured as the percentage of CD4 (**b**) and CD8 (**d**) T-cells with diluted CFSE signal following 96 h stimulation with EfB. S/P = Sample to positive ratio. + = *P* < 0,05 before correction for multiple comparisons. * = *P* < 0,05 after correction for multiple comparisons

The production of IFN-γ and IL17a was measured following stimulation of whole blood with LukM and Efb. After each immunization the IFN-γ production following LukM stimulation rose and there was a trend for a higher production compared to the control group in weeks 7 and 56 (Fig. 4a). IL17a production showed similar dynamics, with a trend for higher IL17a levels at week 7 and significantly higher levels post-partum compared to the control group (Fig. 4c). IFN-γ production following Efb stimulation increased after each immunization, but was not significantly different from the control group (Fig. 5a). Compared to the control group, IL17a production was significantly higher in the experimentally immunized animals in week 7 and there was a trend for higher levels at week 56 (Fig. 5c).

After the prime and first booster immunization, in week 7, there was a trend for a higher proliferative response of CD4 and CD8 T-cells following stimulation with LukM (Fig. 4b, d). Between week 7 and 52 the proliferative response declined in the experimentally immunized animals. Following the second booster immunization, in week 56, there was a trend for a higher CD4 proliferative response and the CD8 response was significantly higher in the experimentally immunized animals. Around parturition the proliferative responses dropped and there were no differences between control and experimentally immunized animals. Differences in proliferative responses between the control and experimentally immunized animals following Efb stimulation were less pronounced (Fig. 5). There was a trend for a higher proliferative response of CD4 T-cells in week 7 (Fig. 5b). No differences in the proliferative response of gamma delta T-cells were found between experimentally immunized and control animals (Additional file 6).

Intracellular expression of IFN-γ and IL17a by CD4 and CD8 T-cells was measured from week 52 onwards. Following stimulation for six days with LukM, both IFN-γ and IL17a levels in CD4 and CD8 T-cells appeared to be higher in the immunized animals in week 56, but this was not significant (Additional file 7a,c,e,g). Intracellular cytokine staining following Efb stimulation showed no effect of experimental immunization (Additional file 7b, d, f, h).

## Discussion

*Staphylococcus aureus* is one of the most common causes of mastitis in dairy cattle and has a huge economical and welfare impact [1, 2], but despite years of research there is currently no effective vaccine [13, 14]. Pre-existing non-protective immunity to *S. aureus* due to natural exposure to *S. aureus* [22–25] may interfere with vaccination. The present study showed that natural exposure to *S. aureus* induced immune responses against *S. aureus* at a very young age, i.e. from 12 weeks of life onwards. Experimental immunization of calves with *S. aureus* immune evasion proteins, starting before natural exposure to *S. aureus,* resulted in significantly higher antibody levels against *S. aureus* immune evasion proteins around parturition in serum, colostrum, and, milk compared to natural exposure.

The neonatal calves enrolled in the first study had no detectible antibodies specific for *S. aureus* before the absorption of maternal antibodies from colostrum. Whereas the maternal antibody levels declined, the rise in antibodies specific for both cell-wall and secreted components of *S. aureus* from week 12 onward showed that the calves mounted an immune response to *S. aureus* following natural exposure at around 3 months of age. Since LukM and Efb are *S. aureus* specific, these antibody responses were not a result of exposure to other Staphylococci. Non-infected animals may acquire *S. aureus* from many different sources, including the environment, animal contact and humans [33, 34]. An immune response against *S. aureus* does not indicate that the calves suffered from *S. aureus* related disease, since many animals have been shown to be non-symptomatic carriers of *S. aureus* on skin and mucosa [33–35]. Nevertheless, the calves did mount an immune response to Efb and LukM, proving that they were exposed to these important virulence factors.

The aim of the experimental immunization study was to test whether immunization of *S. aureus* naïve animals changes the quality and quantity of the immune response compared to natural exposure to *S. aureus*. Calves were housed under the same conditions as the first study group and the first immunization time point was chosen well before the expected rise in *S. aureus* specific antibodies as seen during the first study. The low Efb and LukM specific antibody levels at the start of the experiment and the rise in antibody levels in the ‘natural exposure’ control group between week 7 and week 52 both indicate that the calves were immunized before natural exposure to *S. aureus*. Initially it was intended to compare the immune response following natural exposure to both IN and SC immunization. However, intranasal immunization did not induce a measurable antibody response, which was also seen in a concurrent study using the same experimental vaccine [19]. In contrast to live vaccines [36], recombinant proteins and adjuvant alone may not be enough to stimulate immune responses at the nasal mucosa. After the first boost, immune responses were similar between the IN and Adj groups and, although an effect of the IN or Adj immunization cannot be excluded, it was therefore determined that for the purpose of this study the intranasally immunized animals could be grouped together with the adjuvant group for further analysis as a single natural exposure group. Similarly, immune responses between the IN/SC and SC group were comparable and these groups were also combined into a SC immunization group. Although this meant that for the experimental immunization group half of the calves received three and the other half received four immunizations, no significant difference between the immunized animals were observed (Additional file 2). From this it was inferred that it was valid to compare the experimentally immunized animals as one group to the control animals for the given readouts. After the first booster immunization, there was a tendency for higher antibody levels in the experimentally immunized group, while there were no differences after the second boost. The effect of the second booster may have been obscured by natural exposure to *S. aureus* which (also) resulted in an antibody response in the control group. Additionally, during the second boost the calves had mild to severe respiratory disease, which may have suppressed the response to experimental immunization. The third boost just before parturition did result in significantly higher IgG1 and IgG2 levels specific for Efb and LukM, both systemically (serum) and locally in the udder (colostrum, milk) in the experimentally immunized animals.

Cellular immunity has been shown to be crucial in the defense against *S. aureus.* For cattle, rapid recruitment of neutrophils is believed to be vital in limiting *S. aureus* mastitis [37]. In mice it has been shown that Th1 and Th17 T-cells mediate protection against (intramammary) *S. aures* infections [38–40]. Therefore, cell mediated adaptive immunity against the vaccine antigens was characterized. Overall, the cellular immune responses were low in the experimentally immunized animals. The oil-in-water adjuvant used is known to predominantly induce antibody responses [41] and an alternative adjuvant would have been more suitable to induce robust cell mediated immunity. A tendency for higher IFN-γ production and proliferative response of CD4 and CD8 T-cells specific for LukM of the experimentally immunized animals was observed after the first and second boost. However, after the third boost just before parturition there were no differences between the two groups, which may have been due to the suppression of cell mediated immunity around parturition [42]. In contrast, there was a significantly higher IL17a response to LukM after the third boost in the experimentally immunized cows. Inflammatory responses (to *S. aureus)* in the udder have been associated with IL17a responses [43–46] and neutrophil influx into the udder of cattle immunized and challenged intramammary with ovalbumin correlated with IL17a responses [47]. Th17 cytokines induce proinflammatory and cell mediated immune response and are believed to play an important role in host defense at mucosal sites [48]. Inducing IL17a/Th17 responses may therefore play an important role in protective intramammary immunity against *S. aureus.*

Besides pre-existing non-protective immunity to *S. aureus* due to natural exposure, there may be several other reasons why vaccination against *S. aureus* does not induce a protective immune response. Whereas the udder is the normal site of infection, most vaccines use the parenteral route. Thus, immune cells and antibodies have to pass the blood-udder barrier between the systemic circulation and the udder tissue [32, 49, 50]. Alternative immunization routes and adjuvant formulations may result in increased local immunity [41, 51, 52], but there is limited information of their effects on intramammary immunity in cattle [19, 32, 53]. Inducing local antibody responses in the udder by experimental immunization has led to conflicting results [54–56]. In cattle, local antibody production in the udder is ancillary to translocation of antibodies from serum [32] and parental immunization can increase antibody levels in the milk [19, 53]. Experiments by Rainard et al. [57] demonstrated that parenteral immunization can also lead to strong cellular immune responses in the udder. In the present study, repeated parenteral immunization starting at a young age resulted in high *S. aureus* specific antibodies both in colostrum and milk. Cows are particularly susceptible to intramammary infections during the periparturient period [58] and the high antibody levels in the udder of the experimentally immunized animals both during colostrogenesis (pre-partum) and post-partum indicates that our immunization scheme can boost local immunity during this critical period. Another hurdle in inducing a protective immune response is the production of a wide range of immune evasion factors by *S. aureus* that target both the innate (e.g. phagocytosis, complement) and adaptive (e.g. T-cells, antibodies) arms of the immune system [59–61]. Therefore, although vaccination may induce an immune response against *S. aureus*, this is not necessarily an effective response. We have previously shown that the harmful effects of Efb and LukMF’ can be neutralized by antibodies induced by experimental immunization [19]. Including immune evasion molecules in vaccines may induce antibodies that neutralize their effects, allowing the immune system to effectively target *S. aureus*. The immunization scheme employed here induced high antibody levels specific for *S. aureus* immune evasion molecules at the site of infection. Further research is necessary to examine the functional capacity of the induced antibodies both in vitro and in vivo.

Experimental immunization of *S. aureus* naïve calves may skew the immune response against *S. aureus* towards a protective immune response rather than the non-protective immune response induced by natural exposure. Here it was shown that immunization of *S. aureus* naïve calves resulted in higher antibody levels specific for *S. aureus* immune evasion factors, both systemically and locally in the udder, as compared to animals naturally exposed *S. aureus*. Additionally, immunization induced an IL17a response against LukM, a cytokine that plays an important role in immunity against bacteria in the udder. Experimental immunization of *S. aureus* naïve animals was compared to natural exposure to *S. aureus* only. Follow-up studies comparing different immunization regimens followed by *S. aureus* challenge will be necessary to address whether experimental immunization of *S. aureus* naïve animals alters the quality of the immune response compared to immunization of animals with non-protective immunity due to natural exposure and whether this results in protective immunity against *S. aureus*.

## Conclusions

We showed that it is practically feasible to vaccinate *S. aureus* naïve cattle and that experimental immunization with staphylococcal immune evasion molecules results in an immune response different from that induced by natural exposure to *S. aureus*, leading to significantly higher antibodies specific for *S. aureus* immune evasion factors locally in the udder.

## Additional files

Additional file 1: Comparison of the immune responses against LukM in the intranasal immunization group between week 0 and 7. IgG1 and IgG2 LukM specific antibodies in serum. S/P = Sample to Positive ratio. NS = Not significant. Antibody levels between week 0 and 7 were compared using paired student’s T-test. (PDF 27 kb)

Additional file 2: Comparison of the immune responses against LukM at week 7 between the four initial treatment groups. IgG1 (a) and IgG2 (b) LukM specific antibodies in serum. IFNg (c) and IL17 (d) production following stimulation of whole blood with LukM for 48 h and 72 h respectively. S/P = Sample to Positive ratio. NS = Not significant. Groups were compared using unpaired student’s T-test. (PDF 46 kb)

Additional file 3: Sample dilutions for LukM, Efb and *S. aureus* whole-cell specific IgG1 and IgG2 ELISAs. (PDF 278 kb)

Additional file 4: Flow cytometry gating strategy. (PDF 403 kb)

Additional file 5: Correlation of *Staphylococcus aureus* specific antibodies between colostrum and serum of dams at calving or calves one week after colostrum ingestion. IgG1 (a, c, e) and IgG2 (b, d, f) antibodies specific for whole SA bacterium (a, b), LukM (c, d) and EfB (e, f). Correlation between dam colostrum and dam and calf serum antibody levels was analyzed by linear regression. S/P = Sample to positive ratio. (PDF 56 kb)

Additional file 6: Proliferation of gamma delta T-cells following stimulation with LukM and EfB. Proliferation was measured as the percentage of gamma delta T-cells with diluted CFSE following 96 h stimulation with LukM (a) or EfB (b). + = *P* < 0,05 before correction for multiple comparisons. * = *P* < 0,05 after correction for multiple comparisons. (PDF 29 kb)

Additional file 7: Intracellular cytokine expression of CD4 and CD8 T-cells following stimulation with LukM and EfB. Percentage of CD4 (a, b, e, f) or CD8 (c, d, g, h) T-cells positive for intracellur IFNg (a, b, c, d) or IL17a (e, f, g, h) following 6 day stimulation with LukM (a, c, e, g) or EfB (b, d, f, h). + = *P* < 0,05 before correction for multiple comparisons. (PDF 36 kb)

## Abbreviations

Adj: Adjuvant
Efb: Extracellular fibrinogen-binding protein
FB: FACS buffer
IN: Intranasal application
LukM: leukocidin subunit M
SA: *Staphylococcus aureus*
SC: Subcutaneous immunization
WBC: Total white blood cells
