# Supplementary figures and images for: Immunization of young heifers with staphylococcal immune evasion proteins before natural exposure to Staphylococcus aureus induces a humoral immune response in serum and milk
Source: BMC Vet Res. 2019 Jan 7;15:15. doi: 10.1186/s12917-018-1765-9 (PMC6323680; doi:10.1186/s12917-018-1765-9)

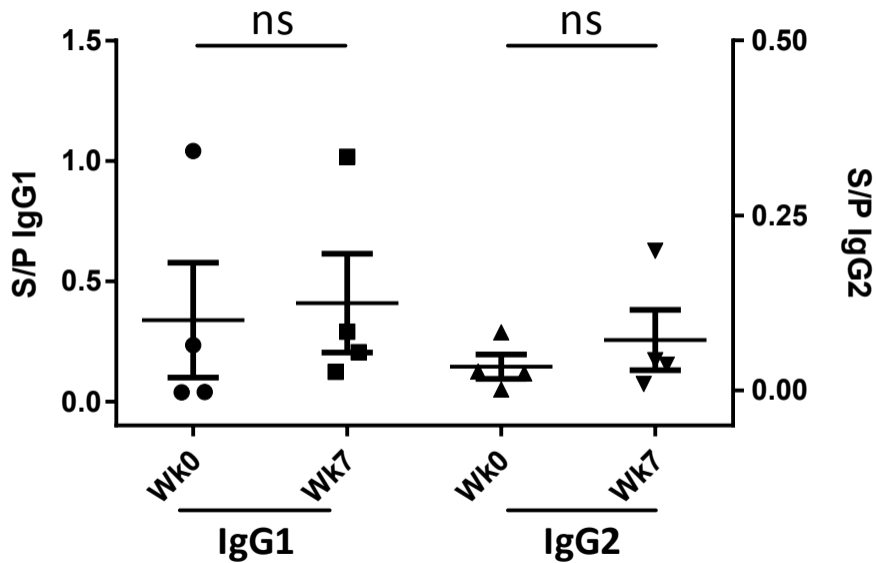

Supplement: Supplementary file 1 — Comparison of the immune responses against LukM in the intranasal immunization group between week 0 and 7. IgG1 and IgG2 LukM specific antibodies in serum. S/P = Sample to Positive ratio. NS = Not significant. Antibody levels between week 0 and 7 were compared using paired student’s T-test. (PDF 27 kb) [file 12917_2018_1765_MOESM1_ESM.pdf]

**A IgG1**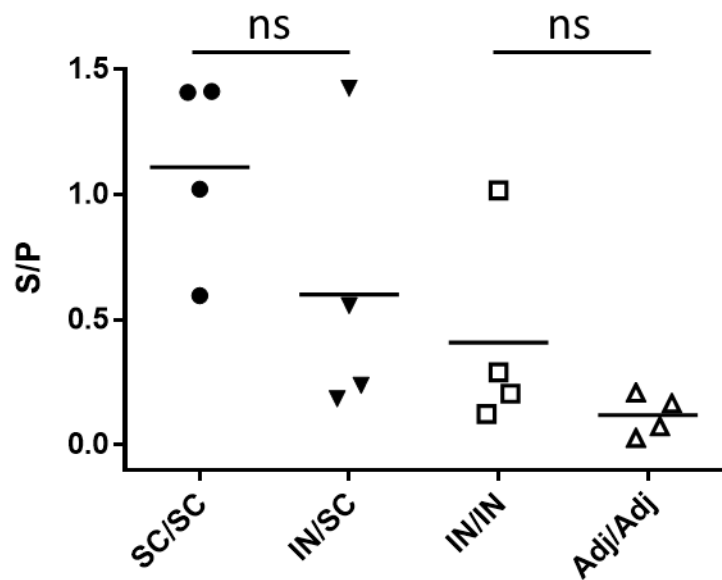**B IgG2**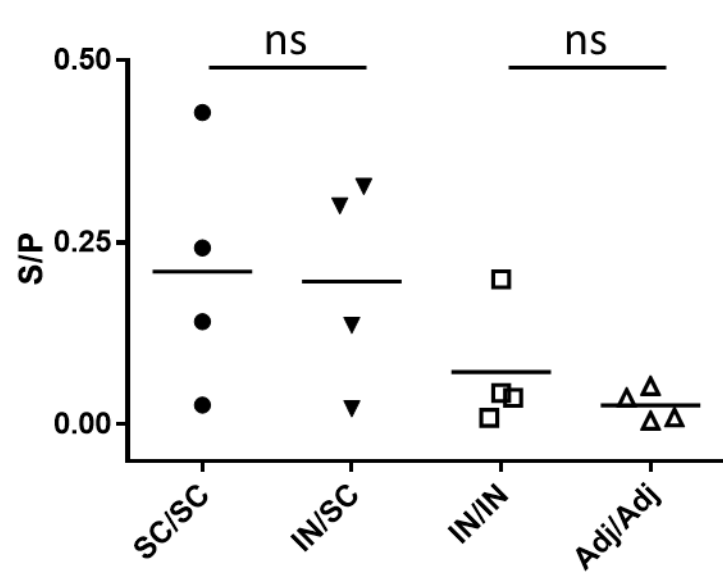**C IFN $\gamma$** 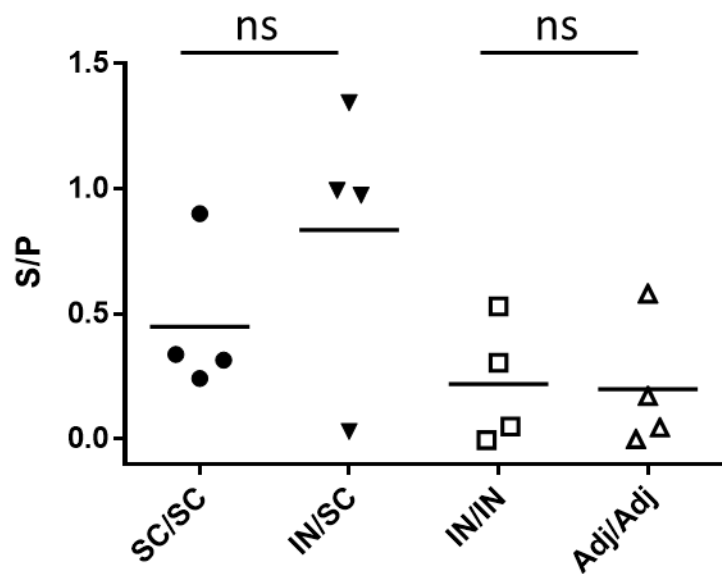**D IL17**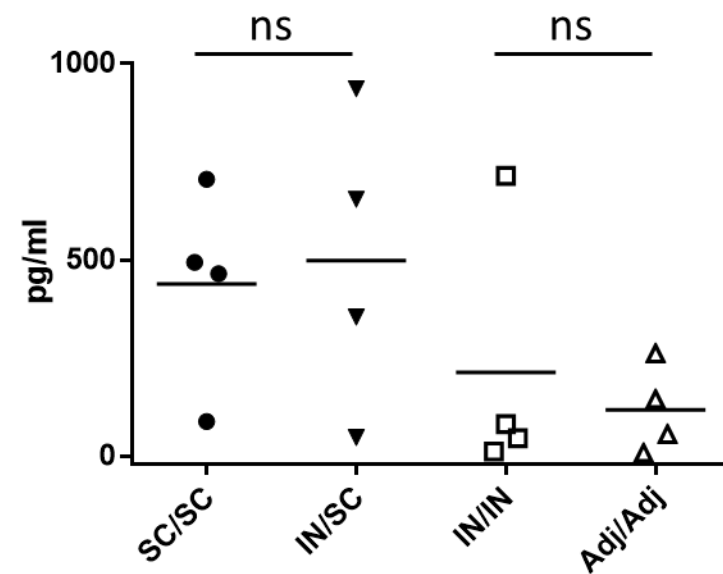

Supplement: Supplementary file 2 — Comparison of the immune responses against LukM at week 7 between the four initial treatment groups. IgG1 (a) and IgG2 (b) LukM specific antibodies in serum. IFNg (c) and IL17 (d) production following stimulation of whole blood with LukM for 48 h and 72 h respectively. S/P = Sample to Positive ratio. NS = Not significant. Groups were compared using unpaired student’s T-test. (PDF 46 kb) [file 12917_2018_1765_MOESM2_ESM.pdf]

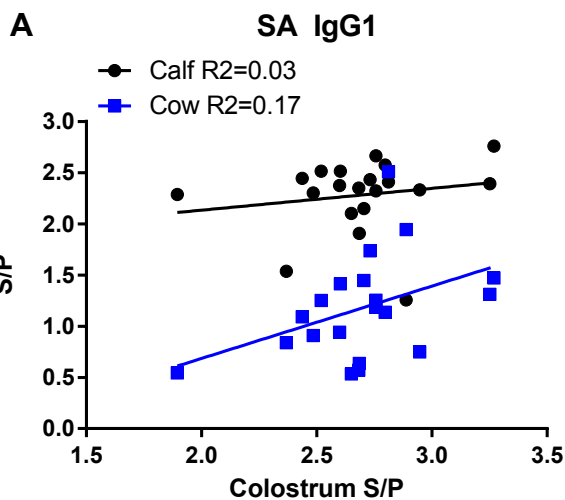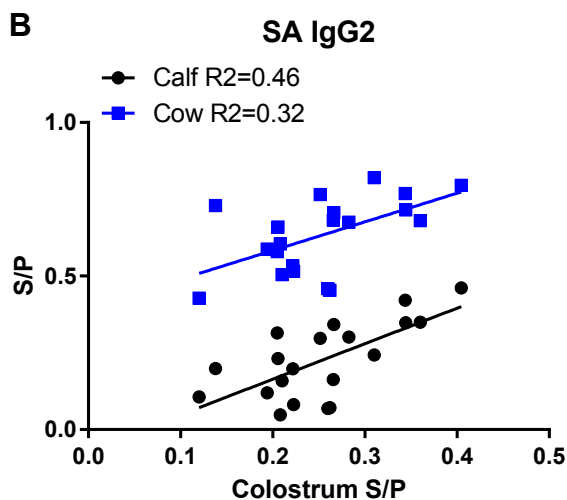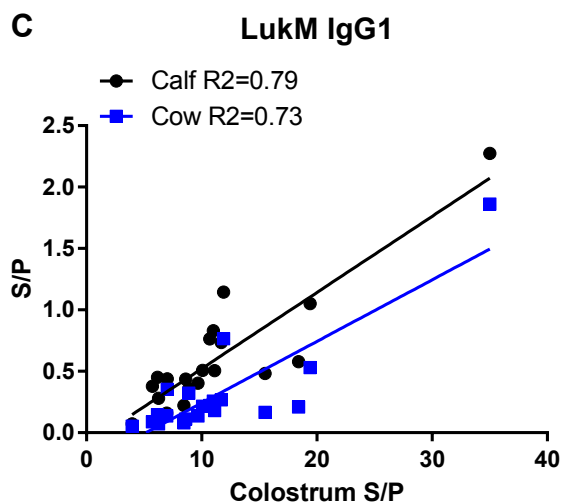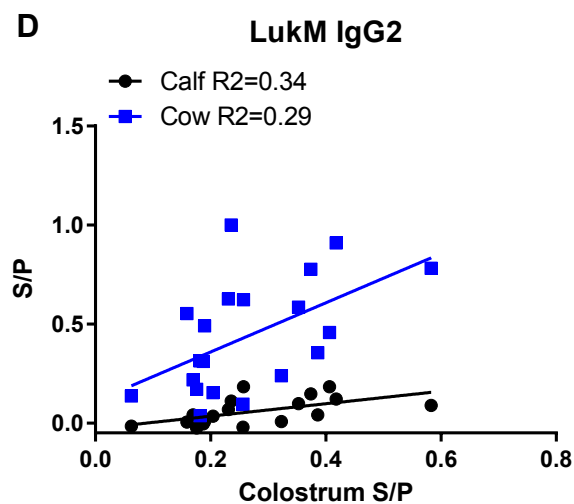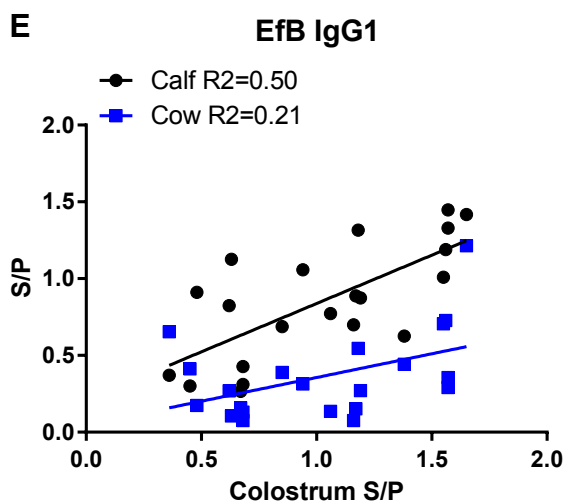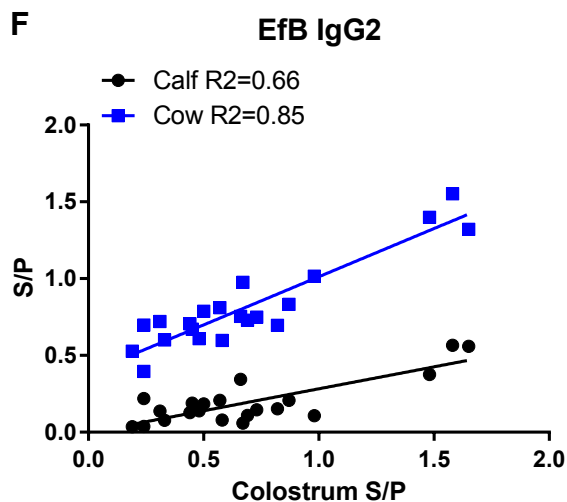

Supplement: Supplementary file 5 — Correlation of Staphylococcus aureus specific antibodies between colostrum and serum of dams at calving or calves one week after colostrum ingestion. IgG1 (a, c, e) and IgG2 (b, d, f) antibodies specific for whole SA bacterium (a, b), LukM (c, d) and EfB (e, f). Correlation between dam colostrum and dam and calf serum antibody levels was analyzed by linear regression. S/P = Sample to positive ratio. (PDF 56 kb) [file 12917_2018_1765_MOESM5_ESM.pdf]

**A**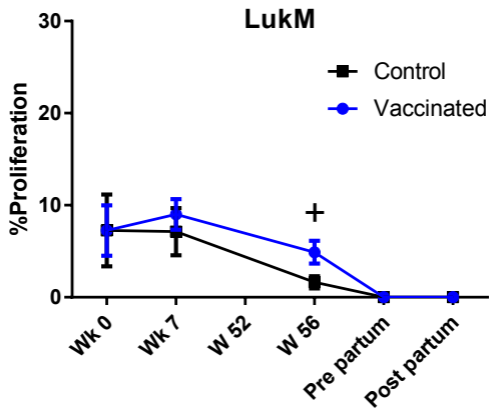**B**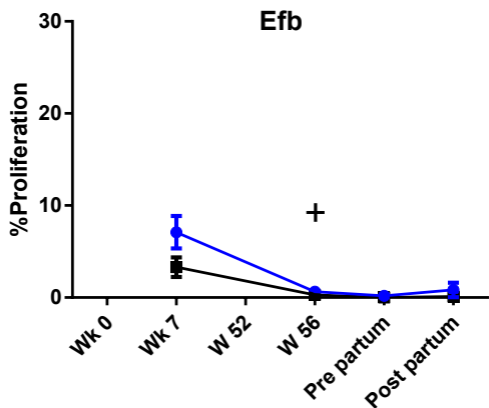

Supplement: Supplementary file 6 — Proliferation of gamma delta T-cells following stimulation with LukM and EfB. Proliferation was measured as the percentage of gamma delta T-cells with diluted CFSE following 96 h stimulation with LukM (a) or EfB (b). + = P < 0,05 before correction for multiple comparisons. * = P < 0,05 after correction for multiple comparisons. (PDF 29 kb) [file 12917_2018_1765_MOESM6_ESM.pdf]

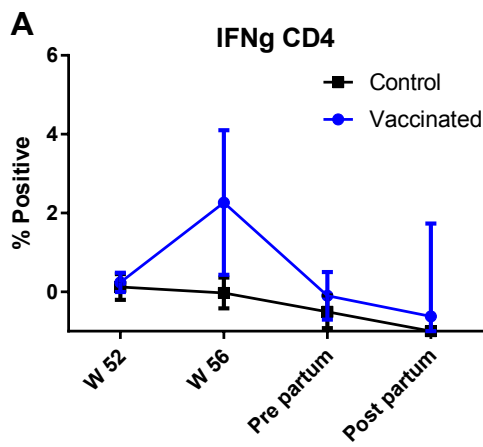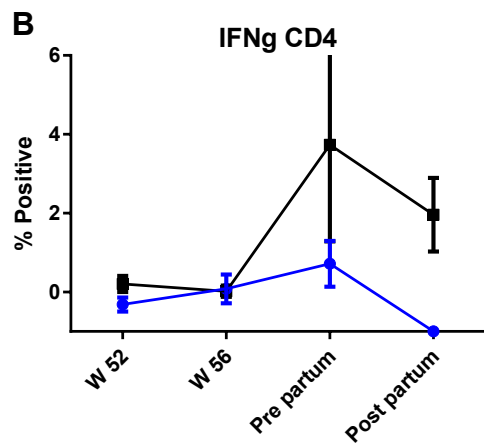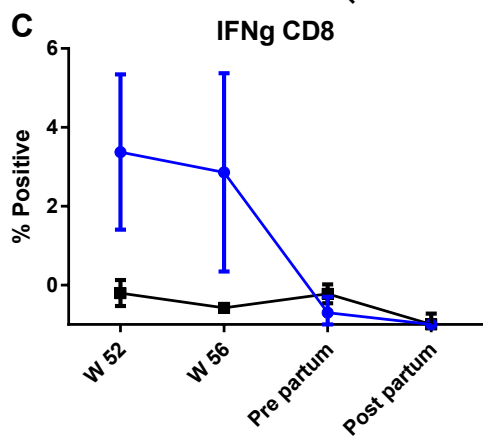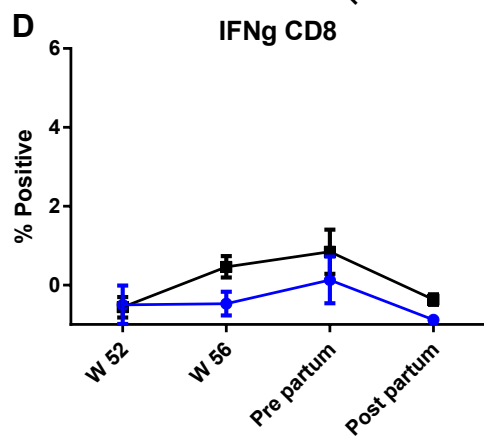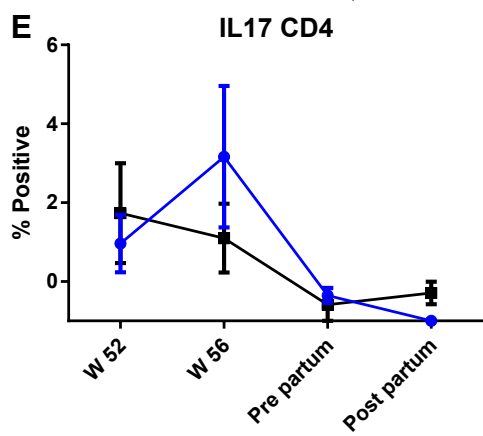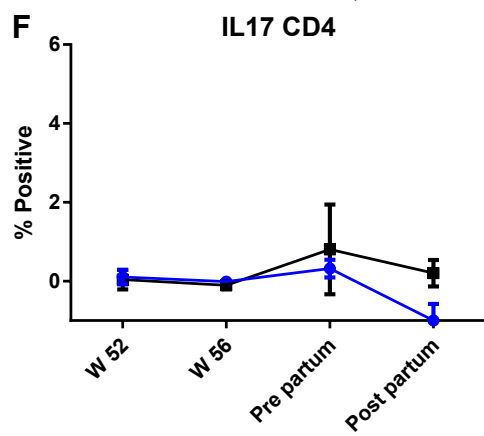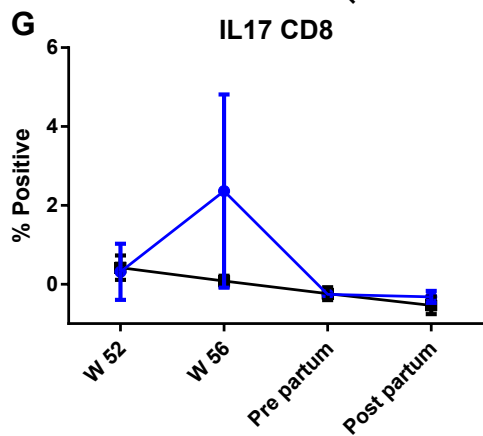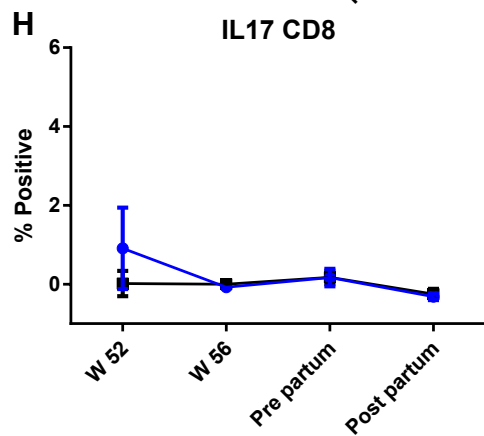

Supplement: Supplementary file 7 — Intracellular cytokine expression of CD4 and CD8 T-cells following stimulation with LukM and EfB. Percentage of CD4 (a, b, e, f) or CD8 (c, d, g, h) T-cells positive for intracellur IFNg (a, b, c, d) or IL17a (e, f, g, h) following 6 day stimulation with LukM (a, c, e, g) or EfB (b, d, f, h). + = P < 0,05 before correction for multiple comparisons. (PDF 36 kb) [file 12917_2018_1765_MOESM7_ESM.pdf]
