# Supplementary material for: Immunization of young heifers with staphylococcal immune evasion proteins before natural exposure to Staphylococcus aureus induces a humoral immune response in serum and milk
Source: BMC Vet Res. 2019 Jan 7;15:15. doi: 10.1186/s12917-018-1765-9 (PMC6323680; doi:10.1186/s12917-018-1765-9)
Supplement: Supplementary file 3 — Sample dilutions for LukM, Efb and S. aureus whole-cell specific IgG1 and IgG2 ELISAs. (PDF 278 kb) [file 12917_2018_1765_MOESM3_ESM.pdf]

Additional file 3. Sample dilutions for LukM, Efb and *S. aureus* whole-cell specific IgG1 and IgG2 ELISAs.

Sample	IgG1<br>Serum	Colostrum	Milk	IgG2<br>Serum	Colostrum	Milk
LukM	1/1000	1/8000	1/10	1/5000	1/1000	1/20
Efb	1/500	1/16000	1/20	1/1000	1/2000	1/80
SA Whole-cell	1/50			1/50		
