# Supplementary material for: Immunization of young heifers with staphylococcal immune evasion proteins before natural exposure to Staphylococcus aureus induces a humoral immune response in serum and milk
Source: BMC Vet Res. 2019 Jan 7;15:15. doi: 10.1186/s12917-018-1765-9 (PMC6323680; doi:10.1186/s12917-018-1765-9)
Supplement: Supplementary file 4 — Flow cytometry gating strategy. (PDF 403 kb) [file 12917_2018_1765_MOESM4_ESM.pdf]

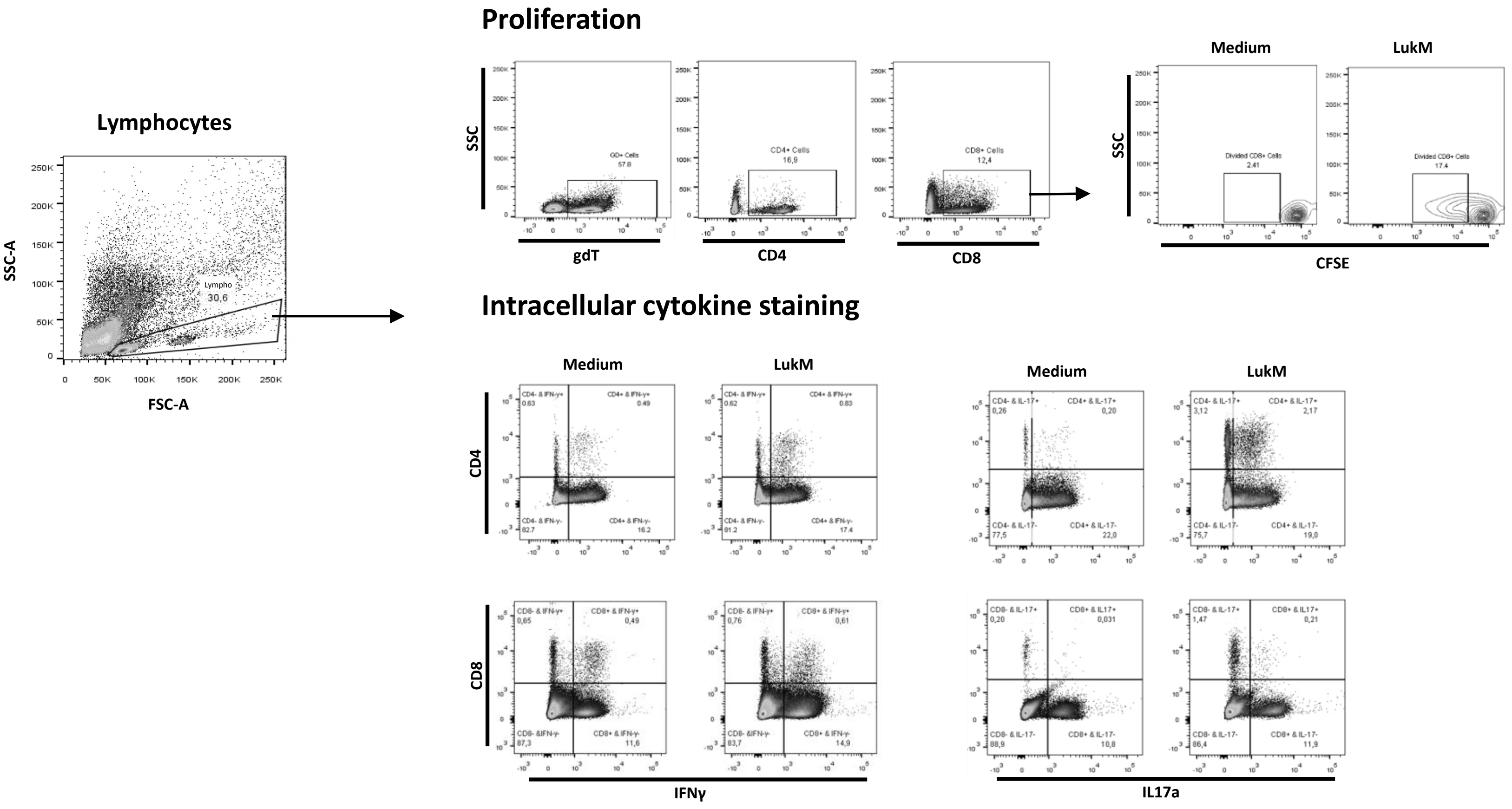

Lymphocytes
SSC-A
FSC-A
Lympho
30,6
Proliferation
SSC
GD+ Cells
57.8
CD4+ Cells
16,9
CD8+ Cells
12,4
gdT
CD4
CD8
Medium
LukM
Divided CD8+ Cells
2.41
Divided CD8+ Cells
17.4
CFSE
Intracellular cytokine staining
Medium
LukM
CD4- & IFN-γ+ 0.63
CD4+ & IFN-γ+ 0.49
CD4- & IFN-γ- 82.7
CD4+ & IFN-γ- 16.2
CD4- & IFN-γ+ 0.62
CD4+ & IFN-γ+ 0.83
CD4- & IFN-γ- 81.2
CD4+ & IFN-γ- 17.4
CD8- & IFN-γ+ 0,65
CD8+ & IFN-γ+ 0,49
CD8- &IFN-γ- 87,3
CD8+ & IFN-γ- 11,6
CD8- & IFN-γ+ 0,76
CD8+ & IFN-γ+ 0,61
CD8- &IFN-γ- 83,7
CD8+ & IFN-γ- 14,9
IFNγ
Medium
LukM
CD4- & IL-17+ 0,26
CD4+ & IL-17+ 0,20
CD4- & IL-17- 77,5
CD4+ & IL-17- 22,0
CD4- & IL-17+ 3,12
CD4+ & IL-17+ 2,17
CD4- & IL-17- 75,7
CD4+ & IL-17- 19,0
CD8- & IL-17+ 0,20
CD8+ & IL17+ 0,031
CD8- & IL-17- 88,9
CD8+ & IL-17- 10,8
CD8- & IL-17+ 1,47
CD8+ & IL17+ 0,21
CD8- & IL-17- 86,4
CD8+ & IL-17- 11,9
IL17a
